# Supplementary material for: On the acceptance, commissioning, and quality assurance of electron FLASH units
Source: Med Phys. 2024 Oct 27;52(2):1207–23. doi: 10.1002/mp.17483 (PMC11788050; doi:10.1002/mp.17483)
Supplement: Supplementary file 1 — Supporting Information [file MP-52-1207-s001.docx]

**Supplementary Material**

**TABLE S1**. Recommendation of useful detectors in ultra-high dose rate electron applications.

| Detector | Application | Reference(s) |
| --- | --- | --- |
| Radiochromic film | Dose measurement, output constancy, energy constancy, linearity, beam profiles, percentage depth dose curves, small field measurements, in-vivo dosimetry | Karsch et al. ^30^  Bazalova-Carter et al. ^32^  Jaccard et. al^46^  Liu et. al^48^ |
| Beam current transformers (BCT) | Output constancy, energy constancy, linearity, pulse discrimination, and potential for real time beam monitoring. | Osterle et. al^43^  Liu et. al^16^ |
| Parallel plate ionization chamber | Output constancy (with recombination mitigation), energy constancy, linearity | Petersson et. al^44^  Gomez et. al^37^   Liu et. al^36^  Kranzer et al.^68^ |
| Diamond detector (adapted for UHDR beams) | Point dose measurement, output constancy, energy constancy, linearity, scanning beam profiles, percentage depth dose curves, small field dosimetry, and pulse discrimination | Marinelli et. al^34^  Verona et. al^64^  Kranzer et al.^69^ |
| Plastic scintillation detectors | Point dose measurement, output constancy, energy constancy, linearity, small field dosimetry, pulse discrimination. | Liu et, al ^63^  Baikalov et al.^61^  Poirier et al.^70^ |
| Luminescent detectors (TLD and OSLD) | In-vivo dosimetry and point dose measurement | Karsch et al.^30^  Liu et. al^49^  Motta et. al^71^  Christensen et al.^72^ |
| Alanine pellets | In-vivo dosimetry and point dose measurement | Gondre et. al^33^ |
| Neutron Detectors | Radiation survey in uncontrolled areas for high energy electron beams | Xiao et al.^73^  Poirier et al.^74^ |


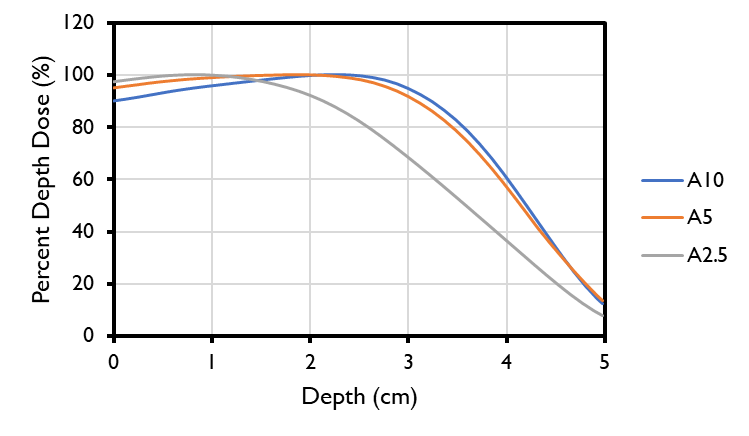


**FIGURE S1.** Percent depth dose (PDD) curves for 9-MeV CONV beam measured for different collimator sizes using the A-cone (A10 is the A cone with a 10-cm collimator; A5 is the A cone with a 5-cm collimator; A2.5, is the A-cone with a 2.5-cm collimator). PDD were measured using a CC04 ionization chamber.
