# Supplementary material for: On the acceptance, commissioning, and quality assurance of electron FLASH units
Source: Med Phys. Author manuscript; Available in PMC 2025 Feb 3. (PMC11788050; doi:10.1002/mp.17483)
Supplement: Supplementary file 1 [file NIHMS2043171-supplement-Supplementary_Material.docx]

**Supplementary Material**

**TABLE S1**. Recommendation of useful detectors in ultra-high dose rate electron applications.

| Detector | Application | Reference(s) |
| --- | --- | --- |
| Radiochromic film | Dose measurement, output constancy, energy constancy, linearity, beam profiles, percentage depth dose curves, small field measurements, in-vivo dosimetry | Karsch et al. ^30^ Bazalova-Carter et al. ^32^ Jaccard et. al^46^ Liu et. al^48^ |
| Beam current transformers (BCT) | Output constancy, energy constancy, linearity, pulse discrimination, and potential for real time beam monitoring. | Osterle et. al^43^ Liu et. al^16^ |
| Parallel plate ionization chamber | Output constancy (with recombination mitigation), energy constancy, linearity | Petersson et. al^44^ Gomez et. al^37^  Liu et. al^36^ Kranzer et al.^68^ |
| Diamond detector (adapted for UHDR beams) | Point dose measurement, output constancy, energy constancy, linearity, scanning beam profiles, percentage depth dose curves, small field dosimetry, and pulse discrimination | Marinelli et. al^34^ Verona et. al^64^ Kranzer et al.^69^ |
| Plastic scintillation detectors | Point dose measurement, output constancy, energy constancy, linearity, small field dosimetry, pulse discrimination. | Liu et, al ^63^ Baikalov et al.^61^ Poirier et al.^70^ |
| Luminescent detectors (TLD and OSLD) | In-vivo dosimetry and point dose measurement | Karsch et al.^30^ Liu et. al^49^ Motta et. al^71^ Christensen et al.^72^ |
| Alanine pellets | In-vivo dosimetry and point dose measurement | Gondre et. al^33^ |
| Neutron Detectors | Radiation survey in uncontrolled areas for high energy electron beams | Xiao et al.^73^ Poirier et al.^74^ |

**FIGURE S1.** Percent depth dose (PDD) curves for 9-MeV CONV beam measured for different collimator sizes using the A-cone (A10 is the A cone with a 10-cm collimator; A5 is the A cone with a 5-cm collimator; A2.5, is the A-cone with a 2.5-cm collimator). PDD were measured using a CC04 ionization chamber.
